# TaClpS1, negatively regulates wheat resistance against *Puccinia striiformis* f. sp. *tritici*

**DOI:** 10.1186/s12870-020-02762-0

**Published:** 2020-12-10

**Authors:** Qian Yang, Md Ashraful Islam, Kunyan Cai, Shuxin Tian, Yan Liu, Zhensheng Kang, Jun Guo

**Affiliations:** State Key Laboratory of Crop Stress Biology for Arid Areas, College of Plant Protection, Northwest A&F University, Yangling, 712100 Shaanxi P. R. China

**Keywords:** *TaClpS1*, *Puccinia striiformis* f. sp. *tritici*, Wheat, Virus-induced gene silencing, Heterologous expression

## Abstract

**Background:**

The degradation of intracellular proteins plays an essential role in plant responses to stressful environments. ClpS1 and E3 ubiquitin ligase function as adaptors for selecting target substrates in caseinolytic peptidase (Clp) proteases pathways and the 26S proteasome system, respectively. Currently, the role of E3 ubiquitin ligase in the plant immune response to pathogens is well defined. However, the role of ClpS1 in the plant immune response to pathogens remains unknown.

**Results:**

Here, wheat (*Triticum aestivum*) ClpS1 (TaClpS1) was studied and resulted to encode 161 amino acids, containing a conserved ClpS domain and a chloroplast transit peptide (1–32 aa). TaClpS1 was found to be specifically localized in the chloroplast when expressed transiently in wheat protoplasts. The transcript level of *TaClpS1* in wheat was significantly induced during infection by *Puccinia striiformis* f. sp. *tritici* (*Pst*). Knockdown of *TaClpS1* via virus-induced gene silencing (VIGS) resulted in an increase in wheat resistance against *Pst*, accompanied by an increase in the hypersensitive response (HR), accumulation of reactive oxygen species (ROS) and expression of *TaPR1* and *TaPR2*, and a reduction in the number of haustoria, length of infection hypha and infection area of *Pst*. Furthermore, heterologous expression of *TaClpS1* in *Nicotiana benthamiana* enhanced the infection by *Phytophthora parasitica*.

**Conclusions:**

These results suggest that TaClpS1 negatively regulates the resistance of wheat to *Pst*.

**Supplementary Information:**

The online version contains supplementary material available at 10.1186/s12870-020-02762-0.

## Background

To ensure their survival in nature, plants must evoke many complicated mechanisms to cope with biotic and abiotic stresses. An increasing number of studies reveal the essential role that the degradation of intracellular proteins plays in plant responses to stressful environments. The protein degradation pathways include ubiquitin–26S proteasome system (UPS) and caseinolytic peptidase (Clp) proteases [1, 2]. The two protein degradation machineries both consist of large multi-subunit proteolytic complexes.

The first process in degradation of proteins by UPS is ATP-dependent ubiquitination, which involves the action of at least three main enzymes for selecting target substrates [3]: ubiquitin-activating enzymes (E1), ubiquitin-conjugating enzymes (E2), and ubiquitin ligases (E3). Studies reveal that ubiquitination during UPS is implicated in many biological processes in plants, including hormone signaling, growth and development, circadian rhythm control and cell cycle [4, 5]. Recent studies have indicated that ubiquitination and E3 ubiquitin ligases are involved in plant immunity to pathogens. For instance, a RING finger E3 ubiquitin ligase, BLAST AND BTH-INDUCED1 (OsBBI1), was found to positively regulate resistance against *Magnaporthe oryzae* by modifying the rice cell wall [6]; SPL1, a rice U-box protein with E3 ubiquitin ligase activity, negatively regulates cell death and innate immunity against *M. oryzae* and *Xanthomonas oryzae* pv. *oryzae* (*Xoo*) [7]; SGT1, an ubiquitin ligase-associated protein, is required for induction of important defense mechanisms, including R gene-mediated defense mechanisms, systemic acquired resistance and basal defense [8]. These studies hinted that factors, which play an important role in selecting target proteins in protein degradation machineries, participate in regulating plant resistance to pathogens. The caseinolytic peptidase (Clp) protease-mediated protein degradation system initially discovered in bacteria consists of a proteolytic protein (mainly ClpP) and some regulatory AAA+ (ATPase associated with diverse cellular activities) proteins [9]. Importantly, regulatory AAA+ proteins use adaptor proteins to recognize and target specific substrates for degradation. For example, in *Escherichia coli*, the regulatory AAA+ protein ClpA uses the adaptor ClpS to recognize and target substrates for degradation by ClpAP protease [10–12]. These studies in *E. coli* indicated that the adaptor ClpS plays a central role in selecting target substrates for degradation by the ClpAP protease, which is the same as the function of E3 ubiquitin ligase in UPS. ClpS from *E. coli* was found to contain two conserved regions by analyzing the crystal structure of ClpS, and they were shown to be involved in the interaction with ClpA and substrates, respectively [13, 14]. Intriguingly, the region involved in the interaction with substrates shared secondary structure homology with E3 ubiquitin ligases in UPS [15]. Moreover, previous phylogenetic analyses of ClpS proteins revealed evolutionary linkages among bacteria, cyanobacteria and plants [15, 16]. Those studies raise the question: could ClpS proteins in plants participate in regulating plant resistance to pathogens just as E3 ubiquitin ligases function in plants?

The plant chloroplast Clp system comprises a hetero-oligomeric protease core complex consisting of five proteolytic subunits (ClpP1 and ClpP3–6) and four different subunits (ClpR1–4), ATP-dependent chaperones ClpC1/2 and ClpD, and an adaptor protein ClpS1, a redefinition of plant ClpS proteins based on subsequent phylogenetic analyses [17, 18]. In *Arabidopsis*, several direct candidate chloroplast AtClpS1 substrates have been identified based on affinity purification methods, including glutamyl-tRNA reductase (GLUTR) and four enzymes in the shikimate pathway [17, 19]. Moreover, the interaction of ClpS1 with the candidate substrates was strictly dependent on two conserved ClpS1 residues involved in recognizing and binding substrates, indicating that ClpS1 is a conserved substrate selector for the chloroplast Clp protease system [17]. In addition, AtClpS1 was reported to also interact with chloroplast chaperones ClpC1, 2 and adaptor ClpF, suggesting a model in which ClpS1 and ClpF form a binary adaptor for selective substrate recognition and delivery to ClpC [17, 18]. However, these reports do not reveal that the plant ClpS1 protein participates in regulating plant resistance to pathogens.

Wheat stripe rust, caused by *Puccinia striiformis* f. sp. *tritici* (*Pst*), is one of the most widespread and destructive diseases of wheat worldwide [20]. In this study, the function of ClpS1 in plant responses to pathogens, based mainly on the interaction system of wheat and the stripe rust pathogen was studied. A *ClpS1* gene from *Triticum aestivum* cv. Suwon 11, designated *TaClpS1* was studied. The transcript level of *TaClpS1* in wheat was induced by the *Pst* isolate CYR23 [21]. Knocking down *TaClpS1* expression in wheat by virus-induced gene silencing (VIGS) attenuated *Pst* infection intensity and enhanced the accumulation of reactive oxygen species (ROS). Moreover, transient expression of *TaClpS1* in *N. benthamiana* facilitated the infection of *Phytophthora parasitica*. These results suggested that TaClpS1 most likely serves as an enhancer of disease in plant which ultimately increases plant susceptibility to pathogen.

## Results

### Identification of the wheat TaClpS1

**Fig. 1:**
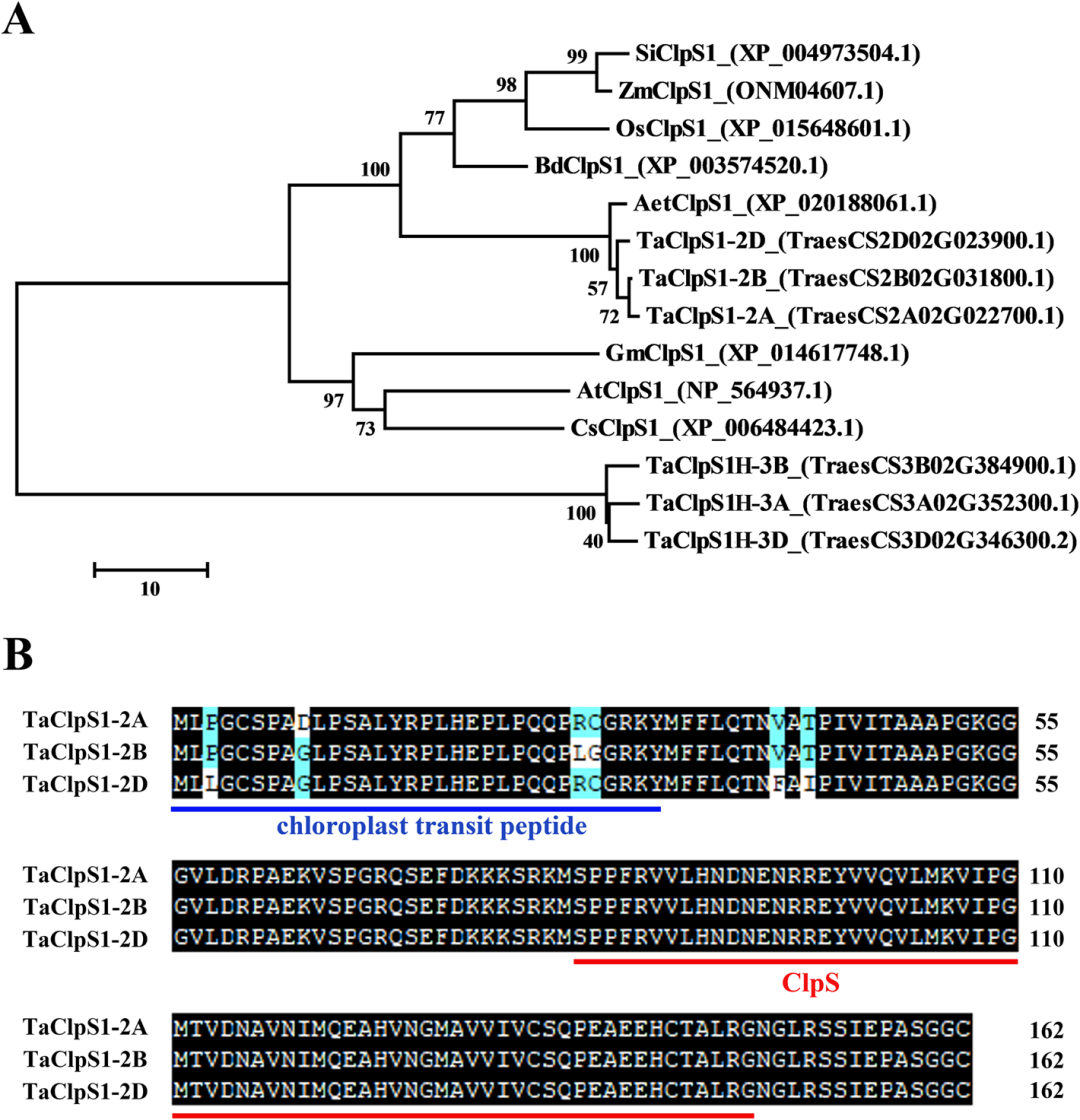
Phylogenetic analysis and protein sequence alignment of TaClpS1. **a** Phylogenetic analysis of ClpS1 proteins from representative species. The phylogenetic tree was developed based on the maximum-likelihood method using MEGA5 software. Ta, *Triticum aestivum*; At, *Arabidopsis thaliana*; Bd*, Brachypodium distachyon*; Os, *Oryza sativa*; Zm, *Zea mays*; Si, *Setaria italica*; *Gm*, *Glycine max*; *Cs*, *Citrus sinensis*; Aet, *Aegilops tauschii*. Protein sequences of TaClpS1 from chromosome 2A, 2B, and 2D of wheat were denoted as TaClpS1-2A, TaClpS1-2B, and TaClpS1-2D, respectively. Sequences from chromosome 3A, 3B, 3D of wheat were denoted as TaClpS1H-3A, TaClpS1H-3B, and TaClpS1H-3D, respectively. **b** Protein sequence alignment of TaClpS1-2A, TaClpS1-2B and TaClpS1-2D. Light blue or colorless shade denotes the amino acid dissimilarities within TaClpS1-2A, TaClpS1-2B and TaClpS1-2D. Regions labelled by red and blue lines represent the ClpS1 domain and the chloroplast transit peptide, respectively

In this study, wheat TaClpS1 was identified using the protein sequence of *Arabidopsis* AtClpS1 (GenBank accession no. NP_564937.1) blasting the hexaploid wheat genome databases (http://plants.ensembl.org/index.html). The results showed that six homologous sequences of AtClpS1 in wheat were located on chromosomes 2A, 2B, 2D, 3A, 3B and 3D, respectively. Phylogenetic analysis of the ClpS1 proteins from various plant species showed that the TaClpS1-2A, TaClpS1-2B, and TaClpS1-2D proteins are closely related to ClpS1 proteins from other plants, including *Arabidopsis*, *Zea mays* and *Oryza sativa*. The TaClpS1H-3A, TaClpS1H-3B, and TaClpS1H-3D proteins are not included in this group (Fig. 1a). Therefore, in this study we focused on the function of TaClpS1-2A, TaClpS1-2B, and TaClpS1-2D.

The predicted proteins TaClpS1-2A, TaClpS1-2B, and TaClpS1-2D all encoded 161 amino acids, with a sequence identity of 98.77%. Sequences of the predicted proteins TaClpS1-2A, TaClpS1-2B, and TaClpS1-2D were determined to contain a conserved ClpS domain by using Pfam online (http://pfam.xfam.org/) (Fig. 1b). Based on the above analyses, it was concluded that the identified genes *TaClpS1-2A*, *TaClpS1-2B*, and *TaClpS1-2D* encode the ClpS1 protein in wheat.

### TaClpS1 is localized in the chloroplast of wheat

**Fig. 2:**
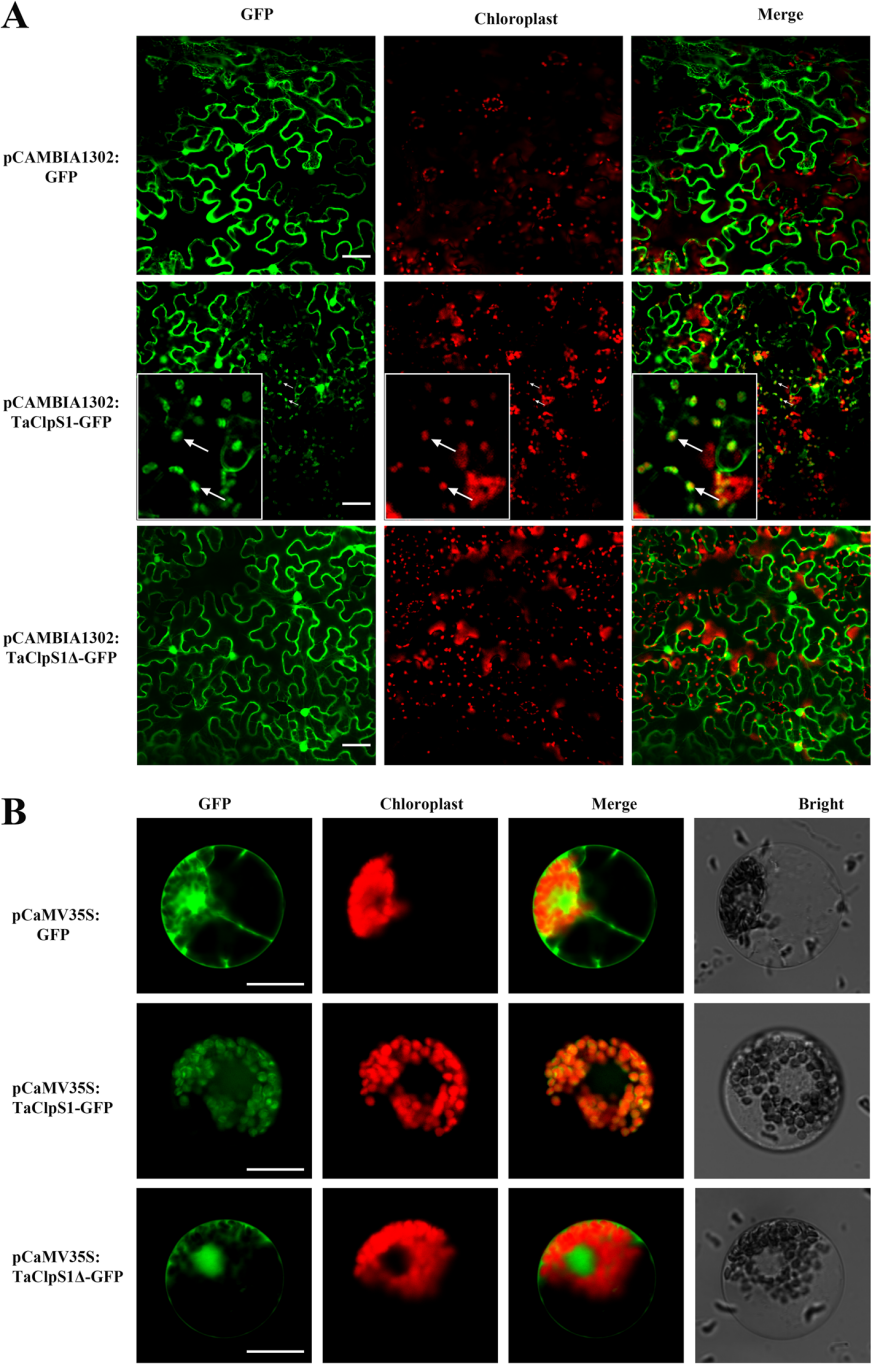
Subcellular localization of TaClpS1 in *Nicotiana benthamiana* and wheat protoplasts*.* **a** Subcellular localization of TaClpS1 in *Nicotiana benthamiana*. pCAMBIA1302: GFP, pCAMBIA1302: TaClpS1–GFP or pCAMBIA1302: TaClpS1Δ–GFP constructs were transiently overexpressed in *N. benthamiana* leaves using agroinfiltration. Olympus IX83 confocal microscope was used to photograph GFP signals at 48 h after agroinfiltration. Bar, 50 μm. **b** Subcellular localization of TaClpS1 in wheat protoplasts. The pCaMV35S: TaClpS1-GFP or pCaMV35S: TaClpS1Δ-GFP or pCaMV35S: GFP constructs were independently overexpressed in wheat protoplasts by PEG-mediated transformation. Bar, 20 μm

To determine the subcellular location of TaClpS1, using localizer online (http://localizer.csiro.au/), TaClpS1-2A, TaClpS1-2B, and TaClpS1-2D were predicted to contain a chloroplast transit peptide (1–32 aa) (Fig. 1b). Considering that TaClpS1-2A, TaClpS1-2B, and TaClpS1-2D are highly conserved in amino acid sequence, TaClpS1-2A was selected as a representative of all TaClpS1 and generated the fusion constructs pCAMBIA1302: TaClpS1–GFP. TaClpS1-2A lacking the chloroplast transit peptide (1–32 aa) (TaClpS1Δ) was fused into vector pCAMBIA1302 to generate pCAMBIA1302: TaClpS1Δ–GFP, which was used as a negative control. These constructs were transformed into *N. benthamiana* leaves via *A. tumefaciens* infiltration. Confocal microscopy showed that TaClpS1–GFP was localized in the nucleus, cytomembrane and chloroplast of *N. benthamiana*, while control pCAMBIA1302: GFP and pCAMBIA1302: TaClpS1Δ–GFP were localized in the nucleus, cytomembrane and cytoplasm (Fig. 2a).

To further confirm the localization of TaClpS1 in wheat cells, the fusion constructs pCaMV35S: TaClpS1-GFP and pCaMV35S: TaClpS1Δ-GFP were generated and transformed into wheat protoplasts by polyethyleneglycol (PEG)-calcium. GFP fluorescence signals of pCaMV35S: TaClpS1Δ-GFP and pCaMV35S: GFP appeared in the nucleus, cytomembrane and cytoplasm of wheat protoplasts. In contrast to the results observed in *N. benthamiana* leaves, GFP fluorescence signals of TaClpS1-GFP aggregated mainly in chloroplasts of wheat protoplasts (Fig. 2b).

### Relative transcript levels of *TaClpS1* at different stages during *Pst* infection

**Fig. 3:**
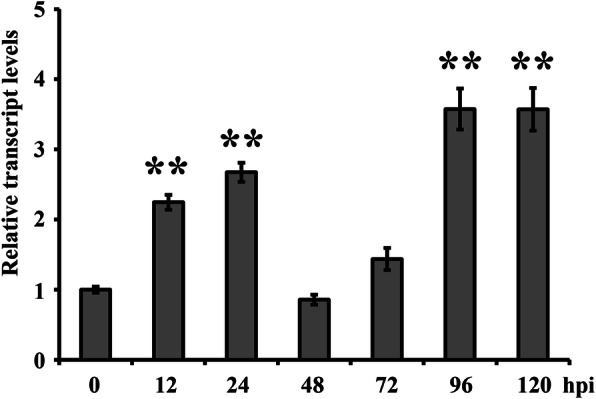
Analysis of *TaClpS1* expression in wheat leaves inoculated with *Pst* CYR23. Wheat leaves (Su11) inoculated with *Pst* CYR23 were sampled at various times, representing the different stages of *Pst* infection. Mock was sampled at 0 hpi. The comparative threshold (2^-ΔΔCT^) approach was used to analyze the relative transcript levels of *TaClpS1*. Data were normalized with the transcript level of wheat elongation factor, *TaEF-1α*, and visualized as the fold changes compared to control at 0 hpi. Each data represent the means ± standard errors of three independent biological replicates. Double asterisks denote the significant difference (*P* < 0.01) from 0 hpi calculated by unpaired two-tailed Student’s *t*-test. Hpi, hours post inoculation

To explore the role of *TaClpS1* during *Pst* infection, the qRT-PCR assay was performed to examine the relative transcript levels of *TaClpS1* at different stages during infection by *Pst* isolate CYR23. qRT-PCR data showed that the transcript levels of *TaClpS1* increased as early as 12 h post-inoculation (hpi), continued to increase to 24 hpi, and subsequently diminished at 48 hpi, before the transcript levels rose again at 96 and 120 hpi (Fig. 3). The qRT-PCR results clearly indicate that the transcript levels of *TaClpS1* in wheat were induced during *Pst* infection, suggesting that *TaClpS1* participates in the interaction between wheat and *Pst*.

### TaClpS1 is a negative regulator of wheat resistance to *Pst*

**Fig. 4:**
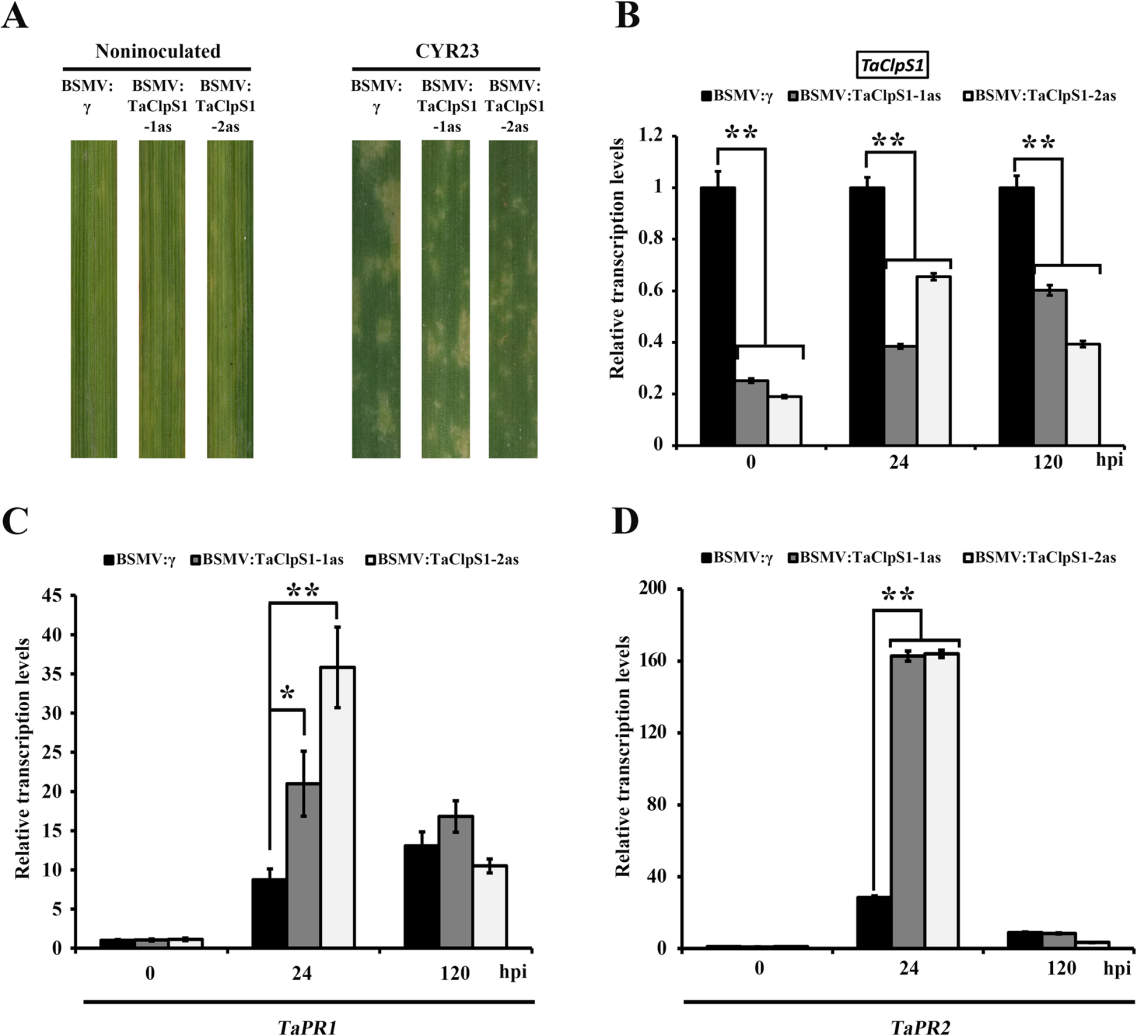
BSMV-mediated silencing of *TaClpS1* enhances wheat resistance to *Pst*. **a** Symptoms of BSMV-infected wheat leaves, and phenotypes of the fourth leaves of silenced plants inoculated with *Pst* isolateCYR23 at 12 dpi. **b**-**d** Relative transcript levels of *TaClpS1* (**b**) and the pathogenesis-related genes*TaPR1* (**c**) and *TaPR2* (**d**) in *TaClpS1*-silenced leaves infected with *Pst* isolate CYR23. The comparative threshold (2^-ΔΔCT^) approach was used to measure the relative transcript levels of *TaClpS1, TaPR1* and *TaPR2*. Data were normalized with the transcription level of *TaEF-1α*, and visualized as the fold changes compared to control at 0 hpi. BSMV: γ infected wheat leaves were used as a control. Each data point represents the means ± standard deviation of three independent biological replicates. Single asterisks (*P* < 0.05) and double asterisks (*P* < 0.01) denote significant differences from BSMV: γ treatment calculated by unpaired two-tailed Student’s *t*-test

**Fig. 5:**
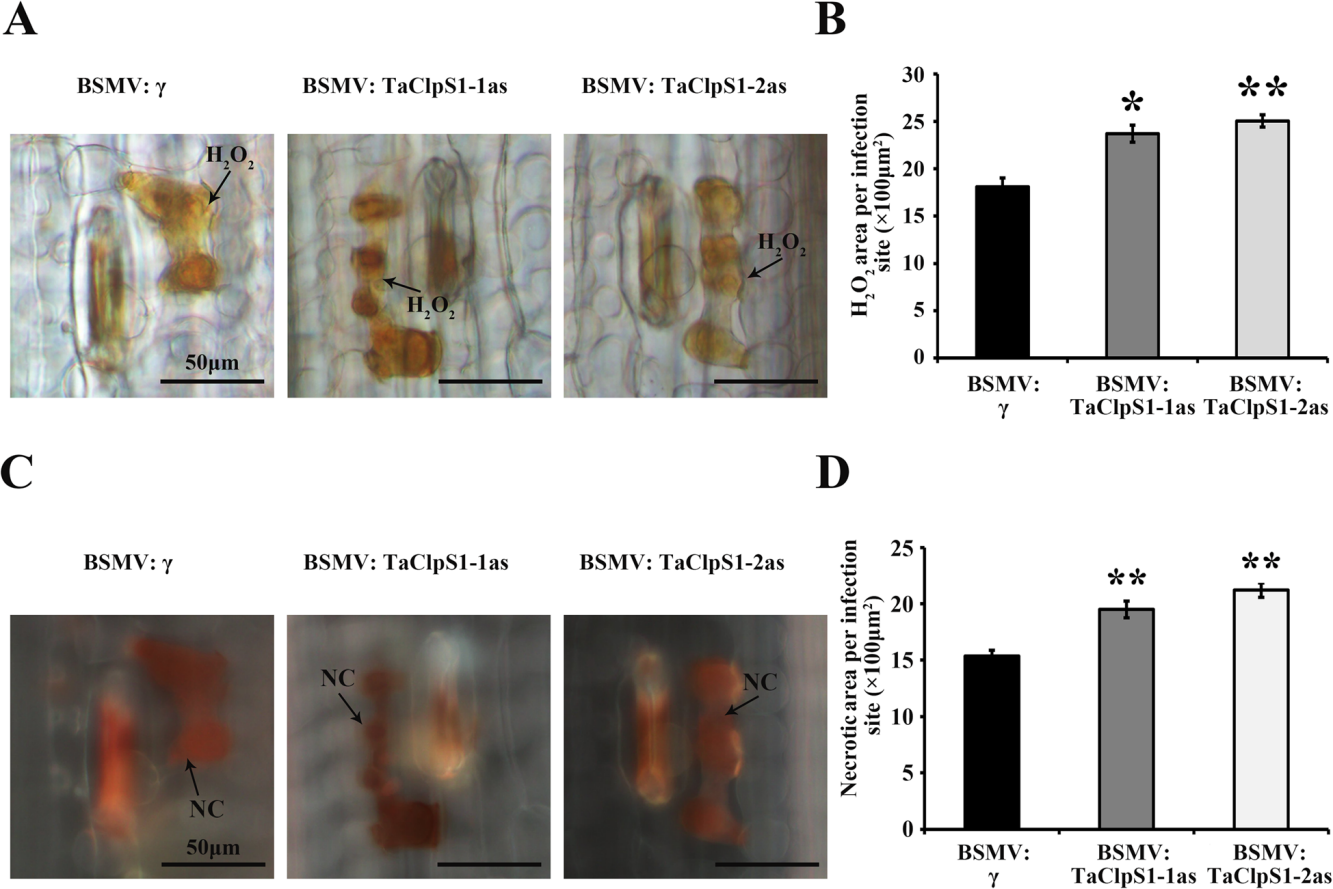
Silencing of *TaClpS1* increases ROS accumulation and necrosis formation on wheat leaves. **a** Wheat plants pre-infected with BSMV: γ or BSMV: *TaClpS1*–1/2as were inoculated with *Pst* isolate CYR23 and stained with 3,3-diaminobenzidine (DAB) for H_2_O_2_ detection at 24 hpi. **b** The amount of H_2_O_2_ production was calculated by measuring the DAB-stained region at each infection site using DP-BSW tools. **c** Necrosis formation was observed microscopically under ultraviolet (UV) light at 24 hpi. **d** Necrotic area per infection site on the wheat leaves treated with BSMV and infected with CYR23 at 24 hpi. NC, necrotic cell. Bar, 50 μm. The 40 successful infection sites and three biological replicates were considered for obtaining all results. Each data denote the means ± standard errors of three biological repetitions. Double asterisks (*P* < 0.01) denote significant differences from BSMV: γ at the same time points calculated by unpaired two-tailed Student’s *t*-test

To examine whether TaClpS1 is involved in regulating the wheat defense resistance against *Pst*, the Barley Stripe Mosaic Virus (BSMV)-induced gene silencing (VIGS) strategy was used. Two specific fragments (*TaClpS1*–1/2as) were designed to specifically silence all three copies of the endogenous *TaClpS1* gene (*TaClpS1-2A*/*2B*/*2D*) in Su11 wheat (Additional file 1: Fig. S1). All the wheat leaves inoculated with BSMV: γ (negative control) or BSMV: *TaClpS1*–1/2as displayed mild chlorotic mosaic symptoms at 12 dpi (Fig. 4a). Subsequently, the fourth leaves of silenced wheat were inoculated with incompatible *Pst* isolate CYR23. The leaves inoculated with CYR23 displayed hypersensitive response (HR) symptoms in negative controls and BSMV: *TaClpS1*–1/2as silenced lines (Fig. 4a). qRT-PCR confirmed that the transcript levels of *TaClpS1* were significantly reduced in BSMV: *TaClpS1*–1/2as silenced lines compared with BSMV: γ treated wheat at 0, 24 and 120 hpi (Fig. 4b). *Pathogenesis-related* (*PR*) genes, including *PR1* and *PR2*, are generally considered as marker genes in HR and are necessary for resistance of plants to pathogens [21, 22]. Then, the transcript levels of *TaPR1* and *TaPR2* were analyzed in TaClpS1–1/2as silenced lines and BSMV: γ treated wheat inoculated with CYR23 at 0, 24 and 120 hpi. Our qRT-PCR results showed that the transcript levels of *TaPR1* (Fig. 4c) and *TaPR2* (Fig. 4d) were notably increased in TaClpS1–1/2as silenced lines compared with that in BSMV: γ treated wheat. In addition, the areas of necroses and H_2_O_2_ accumulation induced by inoculation with CYR23 in wheat leaves were measured at 24 hpi. As shown in Fig. 5, H_2_O_2_ accumulation per infection site (Fig. 5a, b) and the necrotic area (Fig. 5c, d) in TaClpS1–1/2as silenced wheat were obviously greater than that in BSMV: γ treated wheat. Taken together, these results revealed that TaClpS1 stimulates the *Pst* infection in wheat, which finally increased plant susceptibility during wheat-*Pst* incompatible interaction.

### Silencing *TaClpS1* significantly inhibits the growth of *Pst*

**Fig. 6:**
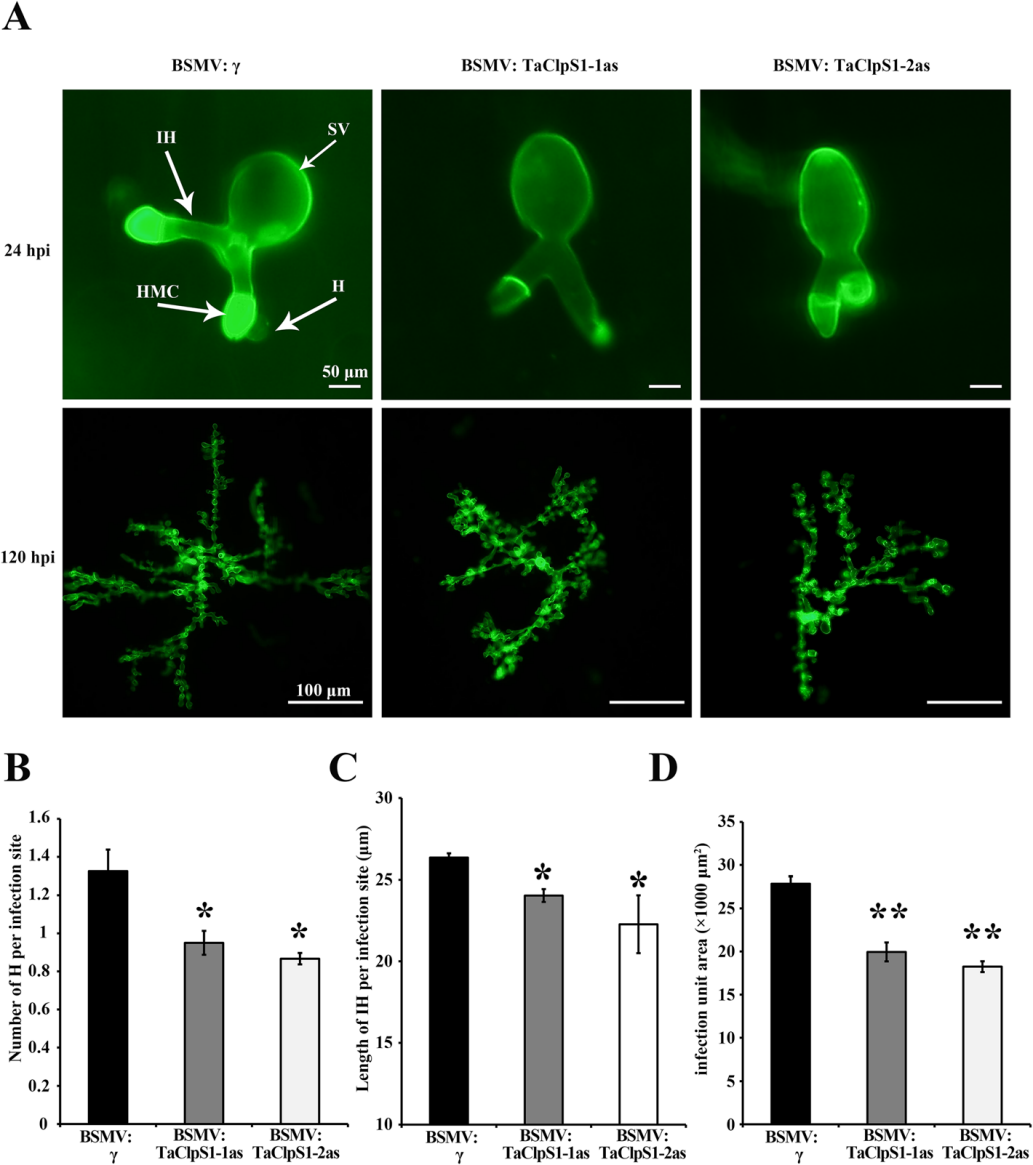
Silencing of *TaClpS1* hinders the expansion of *Pst* on wheat leaves. **a** Wheat germ agglutinin (WGA) was used to stain fungal structures in wheat leaves infected with BSMV and *Pst.* The fungal structures were observed under an autofluorescence microscope. SV, sub-stomatal vesicle; IH, infection hypha; H, haustoria; HMC, haustorial mother cell. **b** The haustoria number per infection site was counted in BSMV-infected wheat leaves inoculated with CYR23 at 24 hpi. **c** The length of IH was measured in BSMV-infected leaves inoculated with CYR23 at 24 hpi. Hyphal length is the distance from the intersection of the sub-stomatal vesicle and the hypha to the tip of the hypha, which was measured by DP-BSW (units in μm) program. **d** The infection area was measured in BSMV-infected leaves inoculated with CYR23 at 120 hpi using DP-BSW program (units in μm^2^). Results shown here were derived from three biological replicates and each replication included 40 individual infection sites. Each data denote the means ± standard deviation of three biological replicates. Asterisks (*P* < 0.05) or double asterisks (*P* < 0.01) indicate significant differences from BSMV: γ treatment at the same time points calculated by unpaired two-tailed Student’s *t*-test

In addition to analyze necroses and H_2_O_2_ accumulation, histological analysis of mycelial structures of *Pst* was performed in wheat leaves infected with CYR23 at 24 and 120 hpi (Fig. 6a). The numbers of haustoria (Fig. 6b), hyphal lengths (Fig. 6c) and hyphal infection area (Fig. 6d), which are indicators to assess fungal expansion ability, were strictly reduced in TaClpS1–1/2as silenced wheat compared with that in BSMV: γ treated wheat. These results indicated that silencing *TaClpS1* diminished the growth of *Pst*.

### TaClpS1 negatively regulates disease resistance of *N. benthamiana* to *Phytophthora parasitica*

**Fig. 7:**
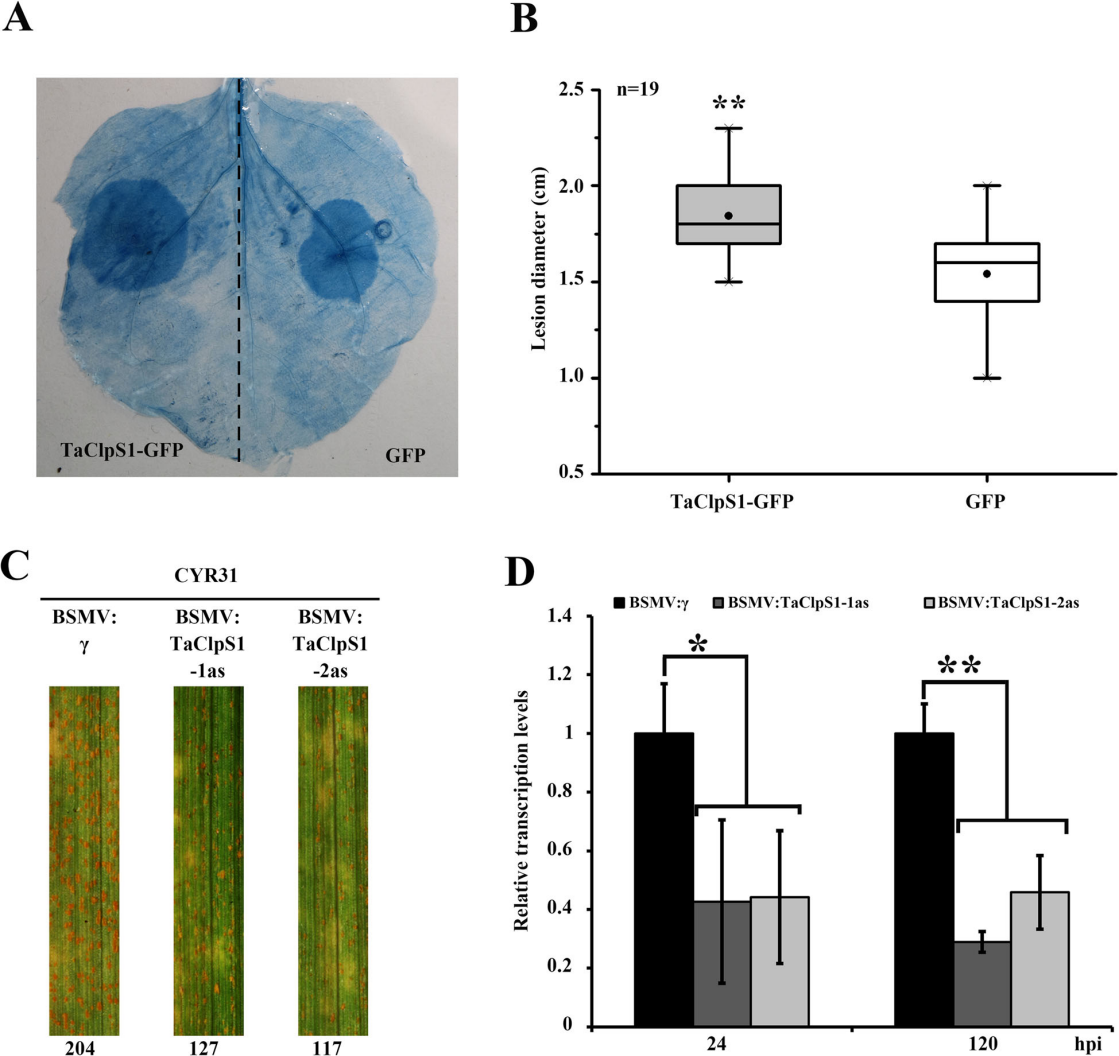
TaClpS1 facilitates the infection of *P. parasitica* and *Pst* in *N. benthamiana* and wheat, respectively. **a** The leaves infiltrated with TaClpS1 or GFP were stained with trypan blue to visualize the colonization of *P. parasitica*. **b** The lesion diameters of *P. parasitica* infection in the leaves infiltrated with TaClpS1 or GFP were analyzed at 36 hpi. Each boxplot illustrates the lesion diameters measured from 19 leaves. The experiment was repeated two times with similar results. The upper quartile, median, and lower quartile are shown in each box, while the bars outside the box indicate the 1st and 99th percentiles. **c** Phenotypes of the fourth leaves of BSMV-infected wheat plants and further inoculated with *Pst* isolate CYR31 at 12 dpi. Numbers below the leaves indicate the number of uredia. **d** Relative transcript levels of *TaClpS1* in *TaClpS1*-silenced plants inoculated with *Pst* isolate CYR31. The comparative threshold (2^-ΔΔCT^) approach was used to measure the relative transcript levels of *TaClpS1*. Data were normalized against the transcription level of *TaEF-1α*, and visualized as the fold changes compared to control at 0 hpi. BSMV: γ infected wheat leaves were used as control. Each data represent means ± standard deviation of three independent biological replications. Single asterisks (*P* < 0.05) or double asterisks (*P* < 0.01) denote significant differences from BSMV: γ treatment calculated by unpaired two-tailed Student’s *t*-test

To further verify the conclusion that TaClpS1 negatively regulates plant resistance against pathogens, firstly the interaction of the model plant *N. benthamiana* and oomycete *Phytophthora parasitica* was examined. In this experiment, *A. tumefaciens* carrying plasmid pCAMBIA1302: TaClpS1–GFP or pCAMBIA1302: GFP (negative control) were infiltrated, and then *P. parasitica* mycelial plugs were placed onto the infiltrated *N. benthamiana* leaves. As expected, compared with the pCAMBIA1302: GFP negative control, lesion diameters of leaves expressing pCAMBIA1302: TaClpS1–GFP were significantly larger, demonstrating that ectopic expression of *TaClpS1* in *N. benthamiana* can enhance *P. parasitica* infection (Fig. 7a, b). In addition, VIGS strategy was utilized to silence *TaClpS1* and then inoculated with *Pst* virulent isolate CYR31. As shown in Fig. 7c, the uredia on *TaClpS1* silenced leaves inoculated with CYR31 at 12 hpi were less than that on negative controls. Moreover, qRT-PCR confirmed that the transcript levels of *TaClpS1* were significantly reduced in BSMV: TaClpS1–1/2as silenced lines compared with BSMV: γ treated wheat at 24 and 120 hpi (Fig. 7d). Taken together, these results indicate that *TaClpS1* negatively regulates disease resistance of plants.

### TaClpS1 interacts with TaHEMA1 by yeast two-hybrid assay

In *Arabidopsis*, GluTR, encoded by gene *HEMA1* (AT1G58290), was identified as a candidate substrate of AtClpS1 [17]. To confirm whether glutamyl-tRNA reductase is a substrate of TaClpS1 in wheat, yeast two-hybrid (Y2H) technique was used. Firstly, a BLAST search using the protein sequence of *Arabidopsis* HEMA1 as a query showed that the hexaploid wheat genome contains three homologous sequences of AtHEMA1, which were located on chromosomes 1A, 1B and 1D, respectively. Subsequently, the three homologous genes were named TaHEMA1 based on phylogenetic tree constructed with HEMA1 proteins from various plant species (Additional file 2: Fig. S2A). Considering that the three copies of TaHEMA1 are highly conserved in amino acid sequence with 98.37% identity (Additional file 2: Fig. S2B), TaHEMA1 in chromosomes 1B was selected as a representative to perform Y2H assay. In Y2H assay, only yeast cell carrying TaHEMA1 and TaClpS1 could grow normally on SD-Leu-Trp-His-Ade medium containing X-α-gal and appeared blue (Additional file 2: Fig. S2C), indicating that TaClpS1 interacts with TaHEMA1. Overall, these results suggest GluTR encoded by *TaHEMA1* could be a candidate substrate of TaClpS1 in wheat.

## Discussion

In this study, the *TaClpS1* was isolated from *T. aestivum* Suwon11 leaves. TaClpS1 is homologous with ClpS1 proteins from various plants, indicating a high sequence conservation of ClpS1 among different plant species. The numbers of uredia, haustoria, hyphal lengths and hyphal infection area, which are indicators to assess fungal expansion ability, were strictly reduced in *TaClpS1*–1/2as silenced wheat compared with that in BSMV: γ treated wheat, suggesting that TaClpS1 promotes susceptibility of wheat to *Pst*.

*Phytophthora* belongs to hemibiotrophic filamentous pathogens, the disease spot size of which in host tissue visually reflects the degree of its infection and suggests the regulation role of host gene to resistance against *Phytophthora*. For instance, the lesion diameter of catalase-deficient *N. benthamiana* leaves inoculated with *P. capsici* was greater than control plants, indicating that catalase positively regulated host resistance against *P. capsici* [22]. Overexpression of *AtRTP5* in *N. benthamiana* leaves resulted in the increase of lesion diameters when inoculated with *P. infestans*, indicating that AtRTP5 plays a negative role in regulating plant resistance against *Phytophthora* [23]. Our results showed that, transient overexpression of *TaClpS1* in *N. benthamiana* enhanced infection of *P. parasitica* (Fig. 7b), suggesting that TaClpS1 promotes susceptibility of *N. benthamiana* to *P. parasitica.*

H_2_O_2_ accumulation area and the average necrotic area per infection site in *TaClpS1*–1/2as silenced wheat were significantly increased, implying that disease resistance was enhanced in *TaClpS1* silenced wheat plants. PR proteins are generally considered as marker genes in HRs and are necessary for resistance [24, 25]. Herein, the transcript levels of *TaPR1* and *TaPR2* were detected in *TaClpS1* silenced wheat plants and negative control infected with the avirulent *Pst* CYR23. At the 0 hpi, there were no significant differences in the transcript levels of *TaPR1* and *TaPR2* in *TaClpS1* silenced wheat plants and negative control, revealing that the transcript levels of *TaPR1* and *TaPR2* were not constitutively induced in non-infected *TaClpS1* silenced wheat plants. At 24 hpi, the transcript levels of *TaPR1* and *TaPR2* were significantly induced in *TaClpS1* silenced wheat and negative controls compared to that at 0 hpi, while the transcript levels of *TaPR1* and *TaPR2* were remarkably greater than that in controls. Considering the fact that the accumulation of PR proteins following a pathogen attack is closely associated with the accumulation of salicylic acid (SA) [26], we speculate that the increase of *TaPR1* and *TaPR2* was accompanied by an increase of SA in *TaClpS1* silenced wheat inoculated with *Pst* CYR23. Additionally, SA, as the representative immune signal, is synthesized in *Arabidopsis* chloroplasts [27, 28], in which TaClpS1 was localized. Taken together, the findings suggest that *TaClpS1* could play a negative role in the SA mediated resistance of wheat to *Pst*.

Glutamyl-tRNA reductase in *Arabidopsis* chloroplast has been identified by affinity purification as a candidate substrate of AtClpS1 [17, 29]. Based on the result that glutamyl-tRNA reductase TaHEMA1 in wheat was shown to interact with TaClpS1 using yeast two-hybrid technique, we inferred that TaHEMA1 may function as a substrate of TaClpS1 in wheat. Glutamyl-tRNA reductase is a control point for tetrapyrrole synthesis [30], and increasing research efforts have revealed that tetrapyrrole biosynthesis is involved in the defense response. For example, tetrapyrrole is the main source of singlet-oxygen generation, and singlet oxygen most likely mediates various biological responses, such as host immunity to pathogens [31]. Taken together, it is reasonable that TaClpS1 interrupts tetrapyrrole synthesis to negatively regulate the response of wheat to *Pst* via selecting glutamyl-tRNA reductase for Clp degration. Future work will be performed to test our hypothesis.

## Conclusions

This study reports for the first time that cloning, localization analysis, and functional characterization of a ClpS1 homolog from wheat of AtClpS1. Expression of *TaClpS1* in wheat was induced during *Pst* infection. Moreover, silencing *TaClpS1* led to a decreased susceptibility of wheat to *Pst*. In addition, heterologous expression of TaClpS1 in *N. benthamiana* enhanced the infection of *P. parasitica*. These results suggest that TaClpS1 negatively regulates the plant resistance to pathogens.

## Methods

### Strains, plant materials and growth

In this study, *Pst* isolates CYR23 and CYR31 were used to investigate the transcript levels of *TaClpS1* and the VIGS assay of *TaClpS1* according to the procedure described previously [32]. Fresh *Pst* urediospores were collected from wheat infected with *Pst*. *P. parasitica* strain ZQ-1 used in this study was routinely maintained on 10% V8 juice medium at 25 °C in the dark [33].

Wheat (*Triticum aestivum* L.) variety Suwon11 (AUS-22519) originating from Seuseun Agricultural Experiment Station (Sariwon, Korea) was registered in the Australian Winter Cereal Collection, Tamworth, Australia. Suwon11, containing *YrSu* resistance gene [34], is resistant to *Pst* isolate CYR23. Suwon11 seedlings were grown and maintained in a climatic chamber at 16 °C. Tobacco (*Nicotiana benthamiana*) plants were grown in growth rooms at 21–25 °C with a 16-h/8-h light/dark cycle. CYR23, CYR31, Suwon11 seeds and *N. benthamiana* seeds were obtained from the Prof. Zhensheng Kang’s Lab (Northwest A&F University, China) [35]. *P. parasitica* strain ZQ-1 was obtained from Prof. Yongli Qiao (Shanghai Normal University, China) [33].

### Plasmid constructs

The full length of *TaClpS1* was cloned into T-simple19 vector to generate TaClpS1-T construct from wheat cultivar Suwon11 cDNA with TaClpS1-specific primers TaClpS1-F/R (Additional file 3: Table S1). To create the constructs for examining the subcellular localization of TaClpS1, full-length *TaClpS1* and *TaClpS1*Δ were amplified from the above TaClpS1-T construct and inserted into pCAMBIA1302 or pTF486 vector [36] to generate pCAMBIA1302: TaClpS1–GFP, pCAMBIA1302: TaClpS1Δ–GFP and pCaMV35S: TaClpS1-GFP, pCaMV35S: TaClpS1Δ-GFP respectively. Primer sequences are reported in Additional file 2: Table S1. For VIGS assay, two approximately 150-bp specific silencing fragments were designed based on the combination of Primer5 and NCBI. Barley stripe mosaic virus (BSMV), is a positive-sense RNA virus with a tripartite genome consisting of RNAs α, β and γ. The two designed fragments were cloned with *Not*I and *Pac*I restriction sites and inserted into original BSMV: γ vector to prepare recombinant plasmids BSMV: *TaClpS1*–1as and BSMV: *TaClpS1*-2as using specific primers shown in Additional file 3: Table S1 [37]. Above all constructs were obtained from the Prof. Zhensheng Kang’s Lab (Northwest A&F University, China) [35].

### Phylogenetic analysis

For phylogenetic analysis of TaClpS1, ClpS1 proteins from other plants were obtained using the protein sequence of *Arabidopsis* AtClpS1 (GenBank accession no. NP_564937.1) to blast NCBI databases. For TaHEMA1, the copies and other related sequences were obtained from the Ensemble Plant database. The phylogenetic tree was constructed using maximum-likelihood method in MEGA5 software. DNAMAN v.7.0 software (LynnonBiosoft, USA) was used to perform multiple sequence alignments and the conserved ClpS domain was analyzed using Pfam online (http://pfam.xfam.org/).

### RNA extraction and analyses of transcript levels

The second leaves of the two-leaf stage wheat seedlings were inoculated with *Pst* isolate CYR23. After inoculation, three independent wheat leaves were sampled at 0, 12, 24, 48, 72, 96, 120 hpi for extracting RNA. Total RNA was extracted with the Quick RNA isolation Kit (Huayueyang Biotechnology, China, Beijing). About 3 μg of the total extracted RNA was used for reverse transcription to cDNA with RevertAid First Strand cDNA Synthesis Kit. For RNA extraction and reverse transcription in VIGS assay of *TaClpS1*, the methods were as described above. LightCycler SYBR Green I Master Mix was used for the qRT-PCR assay, and the transcript levels of genes were normalized to the internal control gene *TaEF-1α*. The primers used in qRT-PCR assay are listed in Additional file 3: Table S1. The statistical significance was evaluated by unpaired two-tailed Student’s *t*-test.

### Subcellular localization analysis

To determine the subcellular localization of TaClpS1 in *N. benthamiana* leaves, *A. tumefaciens* carrying pCAMBIA1302: TaClpS1–GFP, pCAMBIA1302: TaClpS1Δ–GFP or pCAMBIA1302: GFP vector at a final OD_600_ of 0.5 was infiltrated into *N. benthamiana* leaves. Vectors pCAMBIA1302: TaClpS1Δ–GFP and pCAMBIA1302: GFP were used as negative controls. The infiltrated *N. benthamiana* were maintained in growth rooms at 21–25 °C with a 16-h/8-h light/dark cycle. At 48 h after agroinfiltration, confocal images were obtained with an Olympus IX83 confocal microscope (Japan) using excitation wavelength of 488 nm and emission wavelength of 520 nm for GFP, and excitation wavelength of 561 nm and emission wavelength of 640 nm for chloroplast autofluorescence, respectively.

For testing the localization of TaClpS1 in wheat cells, *Triticum aestivum* Suwon11 seedlings were grown in the glasshouse at 25 °C for 2–3 weeks. The fusion constructs pCaMV35S: TaClpS1-GFP, pCaMV35S: TaClpS1Δ-GFP and pCaMV35S: GFP were independently transformed into wheat protoplasts by polyethyleneglycol (PEG)-calcium method as described previously [38, 39]. The mixtures containing pCaMV35S: TaClpS1-GFP or pCaMV35S: GFP and wheat protoplasts were incubated at 22 °C. Images were obtained with an Olympus IX83 confocal microscope (Japan) at 24 h after incubation.

### BSMV-mediated gene silencing

Plasmids BSMV: *TaClpS1*–1as, BSMV: *TaClpS1*-2as and BSMV: γ were linearized followed by transcribing and capping in vitro using the RiboMAX Large-Scale RNA Production System-T7 and the Ribom7G Cap Analog (both by Promega) according to the manufacturer’s instructions. Wheat leaves were inoculated with the capped BSMV transcripts and *Pst* isolate CYR23 or CYR31 according to the procedure described previously [40]. BSMV: γ was used as the negative control. The wheat leaves infected with CYR23 were sampled at 0, 24, and 120 hpi for estimating the transcript levels of *TaClpS1* and *TaPR1/2*, H_2_O_2_ detection, measuring necrotic areas and histological observations in VIGS assay of *TaClpS1*. The symptoms on the wheat leaves were photographed at 12 d after inoculation with *Pst* CYR23 and CYR31. These experiments were repeated at least two times.

### DAB staining for H_2_O_2_ detection, measuring necroses

The wheat leaves inoculated with CYR23 in VIGS assay were sampled and stained in 1 mg/ml 3,3-diaminobenzidine (DAB) solution for 6 h at 16 °C under light. After staining, the leaves were clarified in the destaining solution (absolute alcohol: acetic acid glacial, 1:1) for about one week. Then, the decolored wheat leaves were further clarified with chloral hydrate for two weeks. Subsequently, H_2_O_2_ accumulation in the transparent leaves was detected with an Olympus BX-51 microscope under bright-field. Alongside detection of H_2_O_2_ accumulation, necrotic areas were measured under UV channel. The results were obtained from 40 infection sites. The samples were collected from three independent leaves. The experiments were repeated three times. The statistical significance was evaluated by unpaired two-tailed Student’s *t*-test.

### Histological observations of *Pst* growth

For histological observations of *Pst* growth, the wheat leaves inoculated with CYR23 in VIGS experiments were destained at 24 and 120 hpi in absolute alcohol: acetic acid glacial, 1:1 for about one week. Then, clarified wheat samples were stained with wheat germ agglutinin (WGA) conjugated to Alexa-488 (Invitrogen, USA) as described previously [41, 42]. For each biological replicate, 40 infection sites of each sample from three separate leaves were recorded to assess the number of haustoria, hyphal length and infection area. The statistical significance was evaluated by unpaired two-tailed Student’s *t*-test.

### *P. parasitica* inoculation

*N. benthamiana* leaves infiltrated with *A. tumefaciens* carrying pCAMBIA1302: TaClpS1–GFP, pCAMBIA1302: GFP were detached at 36 h after infiltration and challenged with *P. parasitica* by placing mycelial plugs (5 mm diam). The inoculated leaves were maintained in a growth room at 25 °C in darkness. At 36 hpi, the inoculated leaves were stained with trypan blue as previously described [43]. Stained leaves were photographed and the diameters of the lesion area were measured.

### Yeast two-hybrid assay

The recombinant BD-TaHEMA1 vector was constructed by cloning *TaHEMA1* full length sequence into pGBKT7 with primers TaHEMA1-BD-F/R, and recombinant AD-TaClpS1 vector was constructed by cloning *TaClpS1* full length sequence into pGADT7 with primers TaClpS1-AD-F/R (Additional file 3: Table S1). For interaction assay, BD-TaHEMA1 and AD-TaClpS1 were co-transformed into yeast strain AH109 by the lithium acetate method following Yeast Protocols Handbook (Clontech), and grown on the SD/−Trp-Leu or SD/−Trp-Leu-His selection medium. Colonies from SD/−Trp-Leu-His were picked on SD/−Trp-Leu-His-Ade medium containing X-α-gal again for further selection.

## Supplementary Information

**Additional file 1: Figure S1.** The structure of the two fragments used for silencing *TaClpS1*.

**Additional file 2: Figure S2.** Phylogenetic analysis of TaHEMA1 homologs and interaction of TaHEMA1 and TaClpS1.

**Additional file 3: Table S1.** Primers used in this study.

## Data Availability

All data generated in this study are included in the paper and in the supporting information files. Sequence data of this study are available in the Ensemble Plant database (http://plants.ensembl.org/) under accession numbers TraesCS2A02G022700.1 (TaClpS1) and TraesCS1B02G191200.1 (TaHEMA1), and submitted to NCBI GENEBANK database (https://www.ncbi.nlm.nih.gov/genbank/) under accession numbers MW233893 (TaClpS1) and MW233894 (TaHEMA1).

## Abbreviations

Clp: Caseinolytic peptidase
*Pst*: *Puccinia striiformis* f. sp. *tritici*
VIGS: Virus-induced gene silencing
HR: Hypersensitive response
ROS: Reactive oxygen species
UPS: Ubiquitin–26S proteasome system
E1: Ubiquitin-activating enzymes
E2: Ubiquitin-conjugating enzymes
E3: Ubiquitin ligases
AAA+: ATPase associated with diverse cellular activities
PEG: polyethyleneglycol
qRT-PCR: quantitative real-time PCR
BSMV: barley stripe mosaic virus
hpi: h post-inoculation
PR: pathogenesis-related
WGA: wheat germ agglutinin
DAB: 3,3-diaminobenzidine
