# Supplementary figures and images for: TaClpS1, negatively regulates wheat resistance against Puccinia striiformis f. sp. tritici
Source: BMC Plant Biol. 2020 Dec 10;20:555. doi: 10.1186/s12870-020-02762-0 (PMC7730799; doi:10.1186/s12870-020-02762-0)

**Additional file 1: Figure S1.** The structure of the two fragments used for silencing *TaClpS1*.


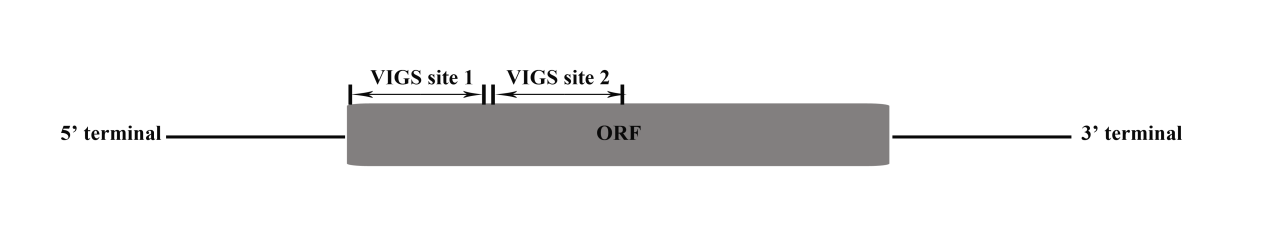

Supplement: Supplementary file 1 — Additional file 1: Figure S1. The structure of the two fragments used for silencing TaClpS1. [file 12870_2020_2762_MOESM1_ESM.docx]
