# Supplementary material for: TaClpS1, negatively regulates wheat resistance against Puccinia striiformis f. sp. tritici
Source: BMC Plant Biol. 2020 Dec 10;20:555. doi: 10.1186/s12870-020-02762-0 (PMC7730799; doi:10.1186/s12870-020-02762-0)
Supplement: Supplementary file 2 — Additional file 2: Figure S2. Phylogenetic analysis of TaHEMA1 homologs and interaction of TaHEMA1 and TaClpS1. [file 12870_2020_2762_MOESM2_ESM.docx]

**Additional file 2: Figure S2.** Phylogenetic analysis of TaHEMA1 homologs and interaction of TaHEMA1 and TaClpS1.


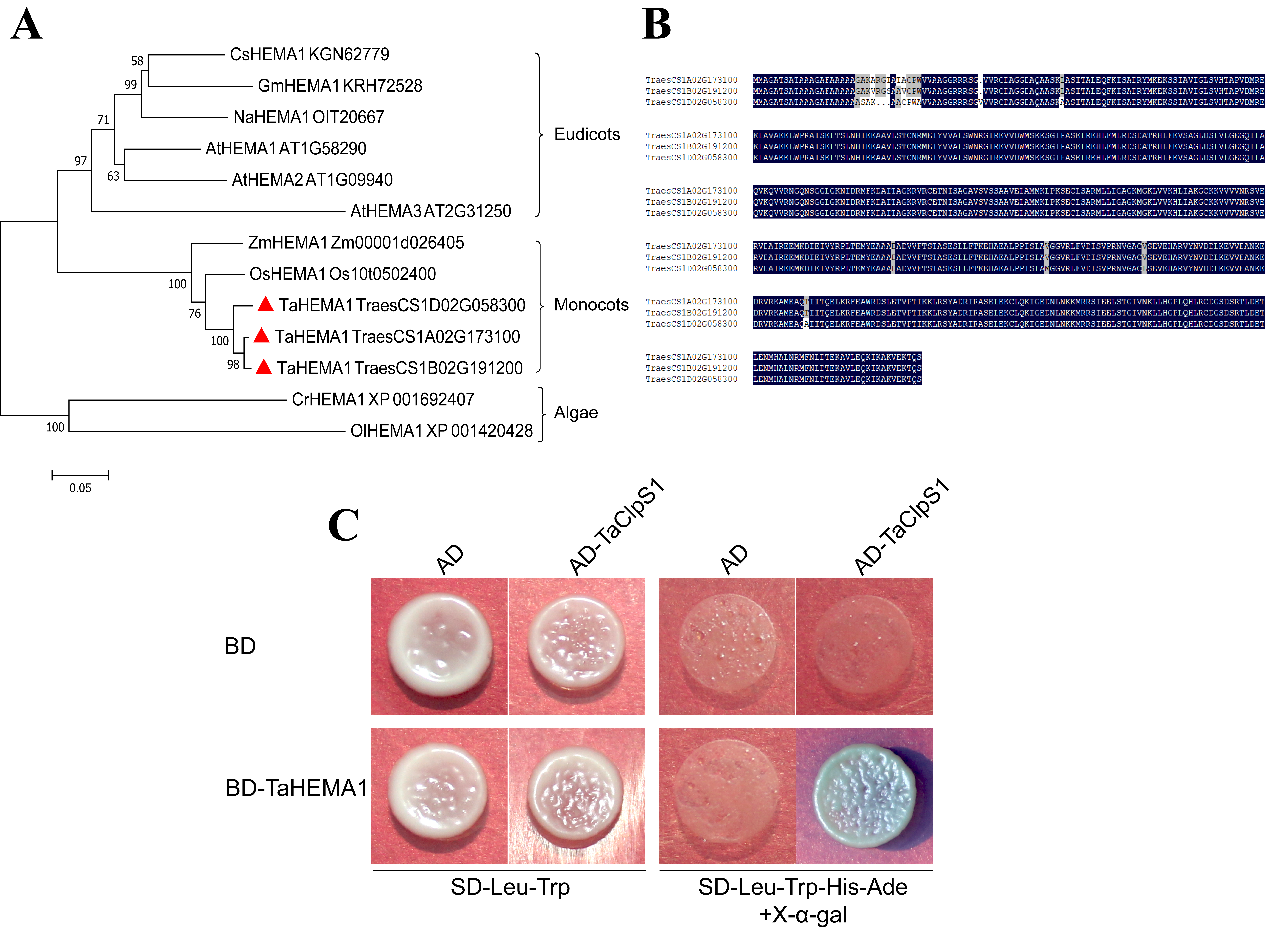


Figure S2. Phylogenetic analysis of TaHEMA1 homologs and interaction of TaHEMA1 and TaClpS1. (A) Phylogenetic analysis of TaHEMA1 and its homologs. MEGA7 software was used to generate the phylogenetic tree by the maximum likelihood method. Branches are labeled with protein names and GenBank accession numbers. Red triangles indicate TaHEMA1 copies in wheat genomes. Ta, *Triticum aestivum*; Os, *Oryza sativa*; Zm, *Zea mays*; Cs, *Cucumis sativus*; Gm, *Glycine max*; Na, *Nicotiana attenuate*; At, *Arabidopsis thaliana*; Cr, *Chlamydomonas reinhardtii*; Ol, *Ostreococcus lucimarinus*. Algae was used as the outgroup. (B) Multiple sequence alignment of the protein sequence of the TaHEMA1 copies. The coding sequences of three TaHEMA1 copies were aligned by DNAMAN software. (C) Y2H interaction analysis between TaHEMA1 and TaClpS1 in yeast. The constructed TaHEMA1-BD interacts with TaClpS1-AD in yeast. Strain AH109 yeast cell containing the indicated pairs of plasmids were grown on selective media SD/-Trp/-Leu or SD/-Trp/- Leu/-His/-Ade containing 20 μg/mL X-α-gal. Plates were photographed 3 days after inoculation. SD, synthetic dropout growth medium.
