# Supplementary material for: TaClpS1, negatively regulates wheat resistance against Puccinia striiformis f. sp. tritici
Source: BMC Plant Biol. 2020 Dec 10;20:555. doi: 10.1186/s12870-020-02762-0 (PMC7730799; doi:10.1186/s12870-020-02762-0)
Supplement: Supplementary file 3 — Additional file 3: Table S1. Primers used in this study. [file 12870_2020_2762_MOESM3_ESM.docx]

**Additional file 3: Table S1.** Primers used in this study.

| **Primers** | **Sequence 5' to 3'** | **use** |
| --- | --- | --- |
| TaEF-qRT-F | TGGTGTCATCAAGCCTGGTATGGT | qRT-PCR primer for internal control gene TaEF1-α |
| TaEF-qRT-R | ACTCATGGTGCATCTCAACGGACT |  |
| qPCRTaClpS1-F | CAATGACAATGAAAACAGGCG | qRT-PCR assay for TaClpS1 |
| qPCRTaClpS1-R | TGCGAGCAGACAATAACTACT |  |
| qPCR-TaPR1-F | GAGAATGCAGACGCCCAAGC | qRT-PCR assay for TaPR1 |
| qPCR-TaPR1-R | CTGGAGCTTGCAGTCGTTGATC |  |
| qPCR-TaPR2-F | AGGATGTTGCTTCCATGTTTGCCG | qRT-PCR assay for TaPR2 |
| qPCR-TaPR2-R | AAGTAGATGCGCATGCCGTTGATG |  |
| TaClpS1-1as-F | CTAGCTGATTAATTAAATGCTGCTAGGATGCTCCCCTG | Construction of BSMV: TaClpS1-1as for BSMV silencing of TaClpS1 |
| TaClpS1-1as-R | CTAGCTGAGCGGCCGCTTTGTCTGTAGAAAGAACATGTA |  |
| TaClpS1-2as-F | CTAGCTGATTAATTAACCCATTGTAATCACAGCAGCTGC | Construction of BSMV: TaClpS1-2as for BSMV silencing of TaClpS1 |
| TaClpS1-2as-R | CTAGCTGAGCGGCCGCCCGGGATTTCTTCTTATCGAAT |  |
| TaClpS1-486-F | CGCGGATCCATGCTGCTAGGATGCTCCCCT | Construction of pTF486-TaClpS1 |
| TaClpS1-486-R | CATGCCATGGGCAGCCACCACTTGCAGGT |  |
| TaClpS1-1302-F | GTAGATCTGACTAGTATGCTGCTAGGATGCTCCCCT | Construction of pCAMBIA1302-TaClpS1 |
| TaClpS1-1302-R | GCTCACCATCCTAGGGCAGCCACCACTTGCAGGT |  |
| TaClpS1Δ-1302-F | GTAGATCTGACTAGTATGTTCTTTCTACAGACAAATTT | Construction of pCAMBIA1302-TaClpS1Δ |
| TaClpS1Δ-1302-R | GCTCACCATCCTAGGGCAGCCACCACTTGCAGGT |  |
| TaClpS1-AD-F | aacatggaggccagtgaattcATGCTGCCAGGATGCTCCC | Construction of AD-TaClpS1 |
| TaClpS1-AD-R | accactgcttgggtggaattcGCAGCCACCACTTGCAGG |  |
| TaHEMA1-BD-F | tcagaggaggacctgcatatgATGATGGCGGGAGCGACG | Construction of BD-TaHEMA1 |
| TaHEMA1-BD-R | tcgacggatccccgggaattcGCTTTGGGTCTTCTCTACCTTGG |  |
